# Determination of the melanin and anthocyanin content in barley grains by digital image analysis using machine learning methods

**DOI:** 10.18699/VJGB-23-99

**Published:** 2023-12

**Authors:** E.G. Komyshev, M.A. Genaev, I.D. Busov, M.V. Kozhekin, N.V. Artemenko, A.Y. Glagoleva, V.S. Koval, D.A. Afonnikov

**Affiliations:** Institute of Cytology and Genetics of the Siberian Branch of the Russian Academy of Sciences, Novosibirsk, Russia; Institute of Cytology and Genetics of the Siberian Branch of the Russian Academy of Sciences, Novosibirsk, Russia Kurchatov Genomic Center of ICG SB RAS, Novosibirsk, Russia Novosibirsk State University, Novosibirsk, Russia; Institute of Cytology and Genetics of the Siberian Branch of the Russian Academy of Sciences, Novosibirsk, Russia Novosibirsk State University, Novosibirsk, Russia; Kurchatov Genomic Center of ICG SB RAS, Novosibirsk, Russia; Kurchatov Genomic Center of ICG SB RAS, Novosibirsk, Russia Novosibirsk State University, Novosibirsk, Russia; Institute of Cytology and Genetics of the Siberian Branch of the Russian Academy of Sciences, Novosibirsk, Russia; Institute of Cytology and Genetics of the Siberian Branch of the Russian Academy of Sciences, Novosibirsk, Russia; Institute of Cytology and Genetics of the Siberian Branch of the Russian Academy of Sciences, Novosibirsk, Russia Kurchatov Genomic Center of ICG SB RAS, Novosibirsk, Russia Novosibirsk State University, Novosibirsk, Russia

**Keywords:** digital image analysis, machine learning, barley grains, pigment composition, анализ цифровых изображений, машинное обучение, зерна ячменя, пигментный состав

## Abstract

The pigment composition of plant seed coat affects important properties such as resistance to pathogens, pre-harvest sprouting, and mechanical hardness. The dark color of barley (Hordeum vulgare L.) grain can be attributed to the synthesis and accumulation of two groups of pigments. Blue and purple grain color is associated with the biosynthesis of anthocyanins. Gray and black grain color is caused by melanin. These pigments may accumulate in the grain shells both individually and together. Therefore, it is difficult to visually distinguish which pigments are responsible for the dark color of the grain. Chemical methods are used to accurately determine the presence/ absence of pigments; however, they are expensive and labor-intensive. Therefore, the development of a new method for quickly assessing the presence of pigments in the grain would help in investigating the mechanisms of genetic control of the pigment composition of barley grains. In this work, we developed a method for assessing the presence or absence of anthocyanins and melanin in the barley grain shell based on digital image analysis using computer vision and machine learning algorithms. A protocol was developed to obtain digital RGB images of barley grains. Using this protocol, a total of 972 images were acquired for 108 barley accessions. Seed coat from these accessions may contain anthocyanins, melanins, or pigments of both types. Chemical methods were used to accurately determine the pigment content of the grains. Four models based on computer vision techniques and convolutional neural networks of different architectures were developed to predict grain pigment composition from images. The U-Net network model based on the EfficientNetB0 topology showed the best performance in the holdout set (the value of the “accuracy” parameter was 0.821).

## Abstract (ru)

Пигментный состав оболочек семян растений влияет на такие важные их свойства, как устойчивость к действию патогенов, прорастание на корню, а также механическая прочность. У ячменя (Hordeum vulgare L.) темная окраска зерен может быть обусловлена синтезом и накоплением двух групп пигментов. Голубая и фиолетовая окраска зерна связана с синтезом антоцианов. Серую и черную окраску придают пигменты меланины. Данные пигменты могут накапливаться в оболочках зерна независимо либо совместно, поэтому визуально определить, накопление каких именно пигментов придает темный цвет зерна, затруднительно. Для точного определения наличия/отсутствия пигментов используются химические и генетические методы, которые дороги и трудоемки. Поэтому создание нового метода для быстрой оценки наличия определенных пигментов в зерновке является актуальной задачей, решение которой поможет при исследовании механизмов генетического контроля пигментного состава зерна. Настоящая работа посвящена разработке метода оценки пигментного состава зерен ячменя на основе анализа цифровых изображений с помощью алгоритмов компьютерного зрения и машинного обучения. Разработан протокол съемки для получения двумерных цифровых цветных изображений зерен. С использованием данного протокола получено 972 изображения для 108 образцов ячменя. Каждый образец мог содержать пигменты антоцианы и/или меланины. Для точного определения содержания пигментного состава образцов применялись химические методы. Для предсказания пигментного состава зерна на основе изображений было разработано четыре модели, основанных на методах компьютерного зрения и сверточных нейронных сетях различной архитектуры. Лучшую производительность на отложенной выборке показала модель сети U-Net, основанная на топологии EfficientNetB0 (значение параметра «точность» составило 0.821).

## Introduction

The color of cereal grain shell is an important trait characterizing the pigments and metabolites contained in it. The presence of pigments in the shell affects various technological properties of the grain (Souza, Marcos-Filho, 2001; Flintham et al., 2002). Grains with dark grain coloration are more coldand drought-tolerant and also have increased resistance to pathogens (Ceccarelli et al., 1987; Choo et al., 2005). Such properties of colored grains are associated with high antioxidant content as well as additional mechanical hardness of grain shells (Ferdinando et al., 2012; Jana, Mukherjee, 2014). The dark color of barley grains occurs due to the synthesis and accumulation of two groups of pigments. Blue and purple coloration of the grain shell is associated with the biosynthesis of anthocyanins. Gray and black color of barley grains is caused by melanin pigment. These two types of pigments can accumulate in the grain shell depending on the genotype both individually and together. Therefore, it is difficult to determine which pigments cause dark grain color by eye.

A number of regulatory genes and genes encoding enzymes involved in pigment biosynthesis control grain shell coloration. Currently, the pathway of anthocyanin biosynthesis has been investigated quite well, but the molecular mechanisms of melanin biosynthesis are still poorly understood (Shoeva et al., 2018; Glagoleva et al., 2020). When studying the mechanisms of genetic control of grain coloration, breeders and geneticists need to assess the pigment content of grain shells. Chemical methods for estimating pigment content allowed to accurately determine the presence/absence of pigments; however, they are expensive and labor-intensive. Other approaches to solving this problem include spectrophotometers, spectrometers, and hyperspectral cameras. However, these cameras are expensive, especially those with high resolution, both spatial and spectral. An alternative is the use of digital RGB cameras that produce high-quality images with high spatial and color resolution (Afonnikov et al., 2016; Li et al., 2020; Kolhar, Jagtap, 2023). In this regard, methods for estimating color and textural characteristics of cereal grains based on the analysis of twodimensional images acquired by digital cameras or scanners have recently been intensively developed in the field of grain phenotyping (Komyshev et al., 2020; Sharma et al., 2021; Afonnikov et al., 2022; Arif et al., 2022; Khojastehnazhand, Roostaei, 2022; Wang, Su, 2022).

The aim of this work is to develop a method for estimating the pigment composition of barley grain based on the analysis of digital images using computer vision and machine learning algorithms.

## Materials and methods

Plant material. Grains of 39 barley accessions with dark colored grain and 40 accessions with light grains were selected for the study. The material was obtained from the barley collection of the All-Russian Institute of Plant Genetic Resources named after N.I. Vavilov (VIR, https://www.vir.nw.ru), the barley collection of the Institute of Cytology and Genetics of the Siberian Branch of the Russian Academy of Sciences (ICG, https://www.icgbio.ru) and the material from the Oregon Wolfe Barleys population (OWB, https://barleyworld.org/ owb). The material description is summarized in Supplementary Material 11. Twenty-nine barley accessions from the VIR collection with different combinations of pigments in the grain were also separately selected (Supplementary Material 2). The material included hulled and hulless barley accessions. 58 hulled and 21 hulless accessions were chosen to create training and test datasets. 22 hulled and 7 hulless accessions were used in the holdout dataset.


Supplementary Materials are available in the online version of the paper: https://vavilov.elpub.ru/jour/manager/files/Suppl_Komyshev_Engl_27_7.pdf


Chemical methods for determining the pigment composition of grains. To determine the presence of anthocyanins in the grain shell, extraction in 1 % HCl solution in methanol, followed by detection of pink coloration of the solution, was performed (Abdel-Aal, Hucl, 1999). The presence of melanin was determined using 2 % NaOH, in which melanin extraction occurs and stains the solution dark (Downie et al., 2003). Based on this method, each of the accessions was assigned a type of pigmentation based on the presence of these pigments (“anthocyanins”, “melanins”) or “no pigments” if both pigments were absent in the grain shell. The presence of pigments of a particular type in the accession seed shells is summarized in Supplementary Materials 1 and 2.

**Fig. 1.**
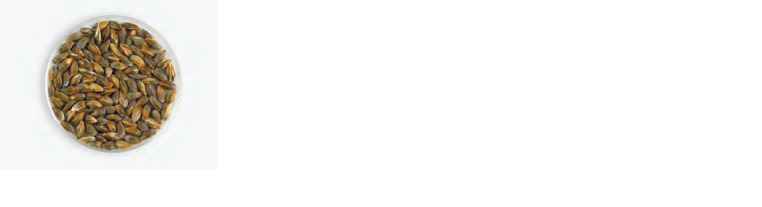
A typical image obtained by the protocol for barley grain phenotyping.

Image acquisition. Color images of grains were obtained using a Canon EOS 600D digital camera, Canon EF 100mm f/2.8 Macro USM lens with a resolution of 18 MP. A 55 mm diameter plastic Petri dish filled with grains without gaps was placed on a white A3 sheet of matte paper. Diffusing light was placed on the sides, and the camera was fixed on a tripod from above, with the lens vertically downward (Supplementary Material 3). Images were saved in JPEG format. Figure 1 shows an example of an image resulting from the protocol.

The Petri dish contained about 100–160 grains. For each accession, 9 images of its replicas were obtained by randomly mixing grains in a Petri dish.

Data markup. In order to develop a segmentation algorithm for 212 images of 59 randomly selected accessions, manual marking of grains and Petri dish boundaries was performed using the LabelMe program (https://github.com/wkentaro/ labelme). An example of a labeled image fragment is shown in Supplementary Material 4. In addition, each image was labeled according to the pigmentation type of the corresponding accession based on experimentally obtained data.

Prediction of grain pigmentation based on machine learning methods. The general scheme for pigmentation type prediction involved segmenting the image into the background and the area occupied by grains and predicting the presence of pigments of a particular type using three methods: (1) a Random Forest algorithm using image color descriptors; (2) a convolutional neural network of the ResNet-18 architecture; and (3) a convolutional neural network of the EfficientNetB0 architecture

Data partitioning scheme for validation and testing. For machine learning methods, the images were divided into three datasets: training (60 % of data: 423 images, 47 accessions); validation (20 % of data: 144 images, 16 accessions); and test (20 % of data: 144 images, 16 accessions). A holdout dataset of 29 accessions including 261 images was used for the final accuracy evaluation. Stratification was used to partition the acquired images (see Supplementary Material 5). Data on the partitioning of the accessions into subsamples are presented in Supplementary Material 5.

Evaluating the accuracy of grain image classification. The output of the trained classification models for each image was represented by two binary numbers, each of which characterized the presence or absence of anthocyanins and melanin. To evaluate the accuracy of the method on the test dataset for each image, the predicted set of such numbers and the true set were compared. The following metrics were calculated based on these comparisons: true positive class predictions (TP), true negative class predictions (TN), total number of positive (P) and negative (N) class representatives. Based on these values, the ACC (accuracy) was calculated according to the formula:

**Formula. 1.**
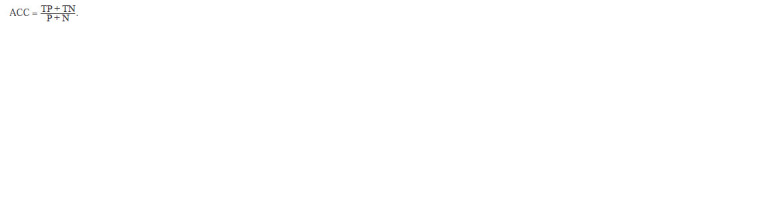
Formula. 1

**Fig. 2.**
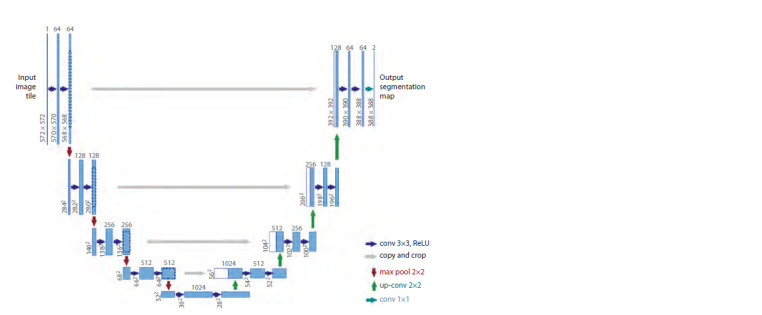
U-Net network architecture used for image segmentation into grain and background regions, from (Ronneberger et al., 2015).

A model for identifying the grain region in an image. To distinguish grains in Petri dishes from the background, the U-Net neural network model with a ResNet-18 encoder was used. The U-Net model was chosen as this architecture had been developed specifically for biomedical image segmentation (Ronneberger et al., 2015). The model is based on the use of convolution and consists of two parts: an encoder and a decoder (Fig. 2). The full-size image at the input of the network is transformed by the encoder through several steps including two consecutive convolution layers of size 3×3 followed by a ReLU transform (labeled as ʻconv 3×3, ReLUʼ layers in Fig. 2) and pooling with a maximum 2×2 function with a step size of 2 (labeled as ʻmax pool 2×2ʼ layers). The encoder performs downsampling of the image. The decoder, on the other hand, performs upsampling of the image using a series of inverse pooling operations that expand the feature map. This is followed by 2×2 convolution, which reduces the number of feature channels (labeled as ʻup-conv 2×2ʼ layers). This is followed by a concatenation with an appropriately edge-cropped feature map from the compressive path and two 3×3 convolutions (labeled as ʻcopy and cropʼ layers in Fig. 2), after each of which a ReLU operation is applied.

Segmentation allowed us to select a region of the Petri dish with grains in the image, which was used to calculate their color descriptors. For each image, 2,380 numerical parameters characterizing the pixel color of the grains were extracted. These are average values of channel intensities for 4 color spaces (RGB, HSV, Lab, YCrCb), values of histograms of color component intensity distributions, etc. Detailed description of the obtained characteristics is given in Supplementary Material 6.

Data filtering. We removed from the prediction input data features, the values of which were identical for all images or did not exceed the value of 0.01 for more than 20 % of images. Additionally, we selected features with pairwise Spearman correlation coefficient less than 0.97 in the image dataset to eliminate redundancy. As a result, 345 color features out of 2,380 remained for our analysis.

Data analysis. In order to estimate the distribution of accessions in the feature space under study, the principal component method (Jolliffe, 2002) and t-SNE algorithm for the nonlinear dimensionality reduction (van der Maaten, Hinton, 2008) were used. These methods allow visualization of multidimensional data by mapping objects from a multidimensional space to a lower dimensional space.

A model for classification of pigment composition of grains based on color descriptors by the Random Forest method

**Fig. 3.**
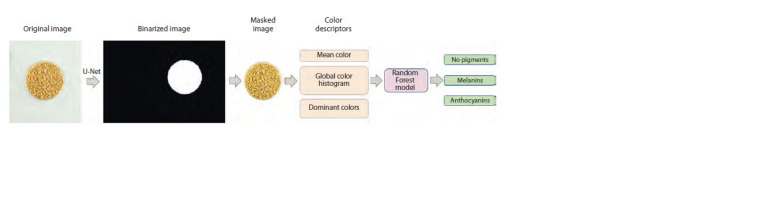
Scheme of the barley grain classification model based on the Random Forest algorithm using color descriptors (the RF13 model).

The classification of grain images into four classes was considered: (1) no pigmentation, (2) presence of anthocyanins only, (3) presence of melanin only, (4) presence of both anthocyanins and melanins. The first classification model was built using the Random Forest algorithm implemented in the Scikit-learn package (Pedregosa et al., 2011). The values of 345 color descriptors described above were used as input. The data processing scheme for this model is shown in Figure 3. Additionally, using the principal component method, the number of features was reduced to 13, which explain 81.2 % of the variance in the data and give the maximum accuracy on the test dataset. We have termed this classification model RF13.

Grain pigment composition classification models based on deep machine learning

ResNet-18 architecture network-based classification model. In addition to the above described RF13 model, three models based on deep machine learning methods were implemented to predict the grain shell pigmentation type. These methods are now widely used to analyze plant images and have been shown to be highly accurate

**Fig. 4.**
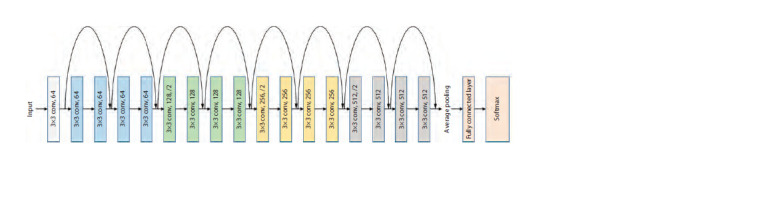
Schematic diagram of ResNet-18 network architecture. Different-colored rectangles show network layers of different structure.

One of the models is the ResNet-18 neural network architecture (He et al., 2016). ResNet is a family of convolutional neural networks (CNNs) of similar architecture differing in the number of layers (18, 34, 50, 101, and 152). In this work, we used a model with 18 layers as the simplest and fastest one. It consists of 17 layers in series including convolution transform, connected by an alternate path for the signal and one full-link layer (Fig. 4). Every four layers, a subsampling operation takes place, where the length and width of the layer becomes 2 times smaller and the number of channels doubles. In Figure 4, these are the layers labeled as “3×3 convolution, N”, where N is the number of channels.

**Fig. 5.**
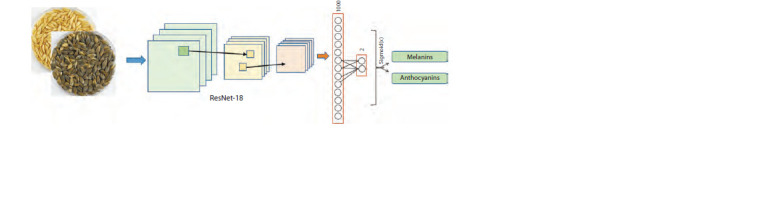
Schematic of the ResNet-18 model of barley grain image classification based on convolutional neural network.

The input of the network was rectangular images, which included regions of Petri dishes (Fig. 5). The output layer included two numbers between 0 and 1 predicting the presence (1) of melanin or anthocyanins. In case the number value was greater than 0.5, the corresponding pigment was considered to be present in the grain shell. This method allowed us to classify images based on the presence of the two pigments in the grains both individually and jointly, and to identify their absence in case both numbers were less than 0.5. This classification model was termed ResNet-18 in our work

**Fig. 6.**
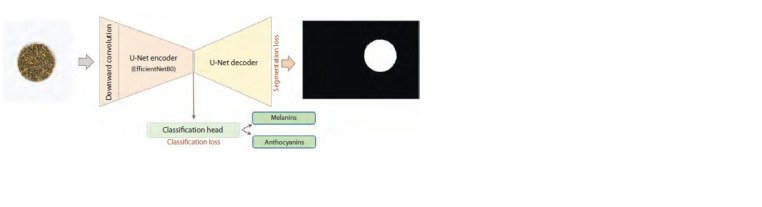
Schematic of the U-Net+ClassHead model based on U-Net segmentation with a head for simultaneous segmentation and classification of barley grain images by the presence/absence of anthocyanins or melanin.

A segmentation-based model with a head for classification. The neural network parameters that were obtained during image segmentation using the U-Net algorithm can be used to classify grains by the presence of pigments. This allows to improve the prediction accuracy for algorithms and to solve two problems simultaneously (segmentation and classification). To this end, an additional output classification layer (“classification head”) was added to the existing segmentationbased model with U-Net architecture (Fig. 6). The output of this layer, as in the ResNet-18 model, contains two numbers to determine the presence of anthocyanins and/or melanin in the grains (see Fig. 6). For this network, the coder topology of the EfficientNetB0 architecture was used (Tan, Le, 2019). This network topology allowed not only to segment the image by selecting a region of grains in a Petri dish on the image, but also to simultaneously perform classification of the whole image based on the presence or absence of the two pigments. This classification model was termed U-Net+ClassHead in the paper

**Fig. 7.**
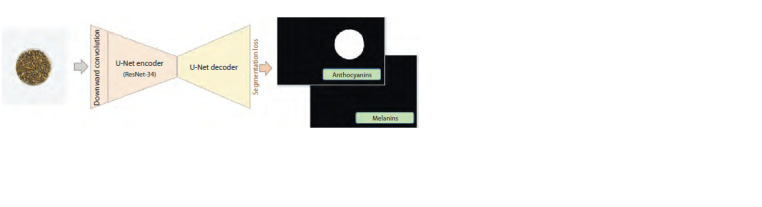
Schematic of the U-Net+ClassSegment model for classification based on 2-channel segmentation of barley grain images by the presence/absence of anthocyanins or melanin.

2-channel segmentation model. For image classification, a modified U-Net can be used to segment each pixel in the image based on the presence of a particular pigmentation. This network outputs a two-channel mask, in which each channel segments the image region if the grain shells contain a particular pigment (Fig. 7). This model, U-Net+ClassSegment, was based on the U-Net architecture with the ResNet-34 encoder. To determine the class of the whole image, we considered that if a single pixel was classified as containing a pigment after segmentation, the whole sample was considered to contain that pigment.

Other technical parameters of training models such as the number of training epochs, batched size, loss function used and optimizer parameters are given in Supplementary Material 7.

**Fig. 8.**
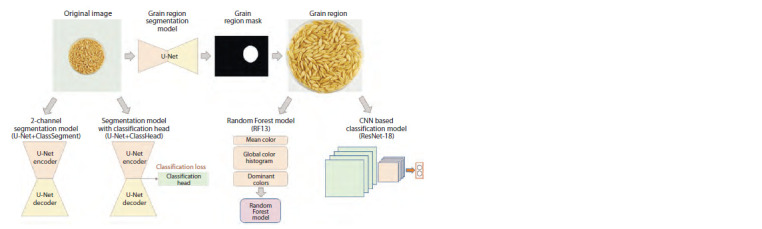
General scheme of barley grain image analysis by the models proposed in this paper

Thus, two classification models based on U-Net segmentation of the original image (U-Net+ClassHead and UNet+ ClassSegment) and two classification models for which the grain region in the original images was separately extracted using the U-Net segmentation model (RF13 and ResNet-18) were considered in this paper. The general scheme of image analysis by the proposed segmentation and classification models is shown in Figure 8.

## Results

Color characteristics of grains

**Fig. 9.**
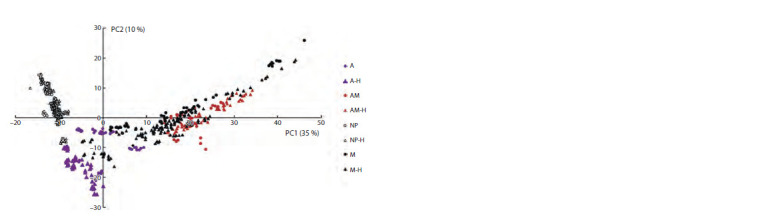
Scattering diagram of the grain images for barley accessions in the space of the first two components derived from PCA for the color characteristics of grains. The X axis is the PC1 component, the Y axis is the PC2 component. Fractions of dispersion for the components are given in parentheses. Grain type designations for pigments and hull presence are shown on the right (A, AM, M, NP – anthocyanins, anthocyanins and melanin, melanin, and no pigments, respectively; H – hulled grains).

**Fig. 10.**
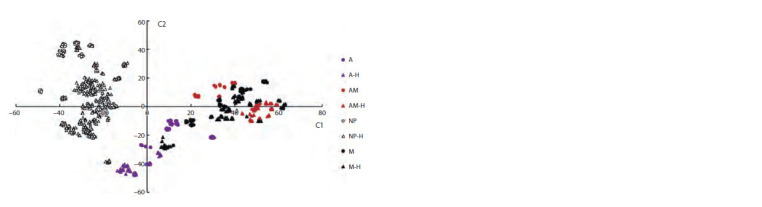
Scatter diagram of the grain images for barley accessions in the space of the first two components resulting from the t-SNE algorithm for the color characteristics of grains. The X axis is the C1 component, the Y axis is the C2 component. Fractions of dispersion for the components are given in parentheses. Grain type designations for pigments and hull presence are shown on the right (A, AM, M, NP – anthocyanins, anthocyanins and melanin, melanin, and no pigments, respectively; H – hulled grains).

PCA and t-SNE methods were applied to map grain images for accessions into a generalized feature space of dimension 2 using 345 informative features (see Materials and methods). The feature values were subjected to normalization before this

analysis (to obtain mean equal to 0 and standard deviation equal to 1). Each point in PCA (Fig. 9) and t-SNE (Fig. 10) diagrams corresponds to a particular image

These diagrams show that pigmented (filled markers) and non-pigmented (empty markers) grains are well separated in both diagrams (see Fig. 9 and 10). This separation is more pronounced in the t-SNE diagram (see Fig. 10). Images of grains with the presence of anthocyanins in the shell (purple icons) and those containing both pigments (red icons) are well separated. The areas occupied by these images in the diagrams do not overlap. At the same time, it is noticeable that the regions occupied by the images of grains with anthocya- nins (filled purple markers) and melanin (filled black markers) overlapped. It is also clear from the diagrams that regions for the images of grains containing both anthocyanins and mela- nin and those containing only melanin have considerable overlap (the right part of the plots close to 0 values for the Y axis). Separating these two types of grains seems most problematic.

The influence the presence of the grain hull has on their color characteristics is also noticeable in the two graphs. First of all, the presence of the grain hull does not affect the separation of areas for different classes of grains by pigmentation except for the pair containing anthocyanins or melanin: hulled and hulless grains with the same type of pigmentation are closer to each other than grains with another type of pigmentation

This is particularly evident for grains without pigmentation (empty markers). For grains with melanin, one of the groups of hulled grains has color characteristics very similar to those of grains with anthocyanins presence (on the graphs, this group is located inside the area occupied by samples with anthocyanins and is far away from other grains containing melanin). At the same time, it is clearly visible that for grains of the same pigment class, hulled and hulless grains occupy different regions and are well separated (characteristic examples in Fig. 10: images of grains without pigmentation, images of grains with anthocyanins, and images of grains with anthocyanins and melanins). These results show that, in most cases, the presence of the hull does not affect separation by the type of grain pigmentation, but significantly affects the variation of shell color characteristics

Classification of grains by pigment content

As a result of training the models to classify grain images by pigment content, accuracy estimates on validation, test and holdout datasets were obtained. They are presented in Table 1.

**Table 1.**
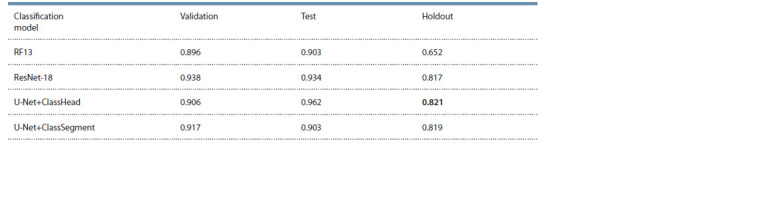
Assessment of classification accuracy (ACC) of barley grain images based on anthocyanins and melanin presence in grain coat for four models on validation, test and holdout datasets Note. The best value for the holdout dataset is shown in bold.

The best accuracy on the holdout dataset is achieved by the segmentation model with “classification head” (U-Net+ClassHead). The data on the parameters of performance estimates of this model are given in Table 2.

**Table 2.**
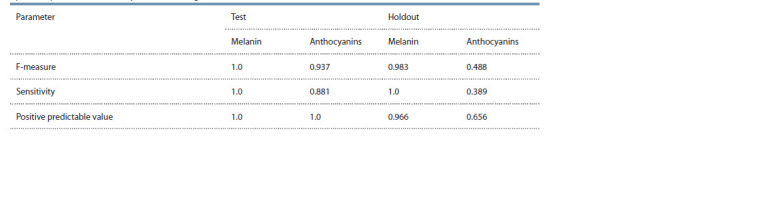
Parameters for evaluation of classification performance of barley grain images by anthocyanins and melanin presence in the grain coat for the U-Net+ClassHead model on test and holdout datasets

The prediction error matrix of the grain pigmentation type (Supplementary Material 8) allows us to determine that most of the model errors are in predicting the anthocyanin content of hulled grains, which is consistent with the PCA and t-SNE plots (see Fig. 9 and 10), where regions for hulled grain images containing melanin and anthocyanins overlap significantly with those containing only melanin. Moreover, the number of images with grain containing anthocyanins (A) predicted as not containing pigments (NP) is significantly larger than the number of images of grains without pigments (NP) predicted as anthocyanins (A). Errors are also observed for hulless grains, for which the presence of anthocyanins was erroneously not predicted. A small number of images of grains with melanins were predicted as “no pigments”, some images of grains containing anthocyanins were identified as containing melanins

The results of the non-parametric Mann–Whitney test showed that the accuracy of anthocyanins presence prediction differs significantly (p-value = 0.004) for hulless and hulled grains. For melanin presence, the hull does not significantly affect the prediction performance

The U-Net+ClassSegment method showed slightly lower accuracy. It can be concluded that models that simultaneously solve several different tasks (multi-task learning) have better generalization ability. Both models based on this approach significantly outperform both the method based on Random Forest and color descriptors (lowest accuracy) and the ResNet-18 classification. It is worth noting that the accuracy results on the holdout dataset are lower than on the test dataset.

## Discussion

Methods for analyzing digital RGB images to study the physiological properties of grains have been widely applied to cereals (Neuman et al., 1989; Huang et al., 2015; Sabanci et al., 2017; Kozłowski et al., 2019; Komyshev et al., 2020; Zykin et al., 2020). In particular, they are used to classify grains both by pigment composition and by variety.

In our work, we analyzed methods for classifying grains by color characteristics into classes based on the presence of two types of pigments. We showed that deep machine learning methods yield higher accuracy in grain classifica- tion than using color descriptors. Similar findings were obtained when classifying barley grains into species (Kozłowski et al., 2019). Our results also show that using a multi-task learning approach produces more accurate classification results.

The results on the holdout image dataset showed lower accuracy compared to the test dataset. Presumably, one of the reasons for this could be that the balance of labels of different classes in the training, validation, and test datasets was the same and was not close to the ratio in the holdout dataset. In particular, the number of images with grains without pigments in the holdout dataset was 1.5 times lower than in the training sample. For classification, such an image set appears to be the easiest case. Also, based on the extracted color descriptors, a binary classifier was trained that distinguished grains from the holdout dataset from other grains with ACC = 1. This implies that there are significant differences between these image series, which can be explained by the fact that grains from other collections were selected in the holdout dataset or the protocol for capturing these images was slightly different. This can explain the slight decrease in accuracy in the classification quality of the Random Forest model

Our analysis also demonstrated that the presence of the hull affects grain color characteristics and, thus, the classification performance with respect to the pigment presence in the shell.

## Conclusion

The proposed methods based on the analysis of digital images using computer vision and machine learning algorithms showed acceptable classification ability in the task of determining melanin and anthocyanins presence or absence in the barley grain shell. The results of this work showed that the application of the Random Forest algorithm based on color features is inferior to convolutional neural network approaches in the classification performance. This method proves to be sensitive to small changes in protocol or imaging conditions, losing generalization ability compared to convolutional neural networks. Possible ways to improve the model based on this algorithm are careful selection of features and preliminary normalization of the images fed to the input. The classical classification model architecture is inferior in accuracy to the 2-channel whole image segmentation model. Segmentation by U-Net neural network with “classification head”, showed the best results (ACC = 0.821) and is the preferred choice in the task of determining the pigment content of barley.

## Conflict of interest

The authors declare no conflict of interest.

## References

Abdel-Aal E.S.M., Hucl P. A rapid method for quantifying total anthocyanins
in blue aleurone and purple pericarp wheats. Cereal Chem.
1999;76(3):350-354. DOI 10.1094/CCHEM.1999.76.3.350

Afonnikov D.A., Genaev M.A., Doroshkov A.V., Komyshev E.G.,
Pshenichnikova
T.A. Methods of high-throughput plant phenotyping
for large-scale breeding and genetic experiments. Russ. J. Genet.
2016;52(7):688-701. DOI 10.1134/S1022795416070024

Afonnikov D.A., Komyshev E.G., Efimov V.M., Genaev M.A., Koval
V.S., Gierke P.U., Börner A. Relationship between the characteristics
of bread wheat grains, storage time and germination. Plants.
2022;11(1):35. DOI 10.3390/plants11010035

Arif M.A.R., Komyshev E.G., Genaev M.A., Koval V.S., Shmakov
N.A., Börner A., Afonnikov D.A. QTL analysis for bread wheat
seed size, shape and color characteristics estimated by digital image
processing. Plants. 2022;11(16):2105. DOI 10.3390/plants11162105

Ceccarelli S., Grando S., Van Leur J.A.G. Genetic diversity in barley
landraces from Syria and Jordan. Euphytica. 1987;36(2):389-405.
DOI 10.1007/BF00041482

Choo T.M., Vigier B., Ho K.M., Ceccarelli S., Grando S., Franckowiak
J.D. Comparison of black, purple, and yellow barleys. Genet.
Resour. Crop Evol. 2005;52(2):121-126. DOI 10.1007/s10722-003-
3086-4

Downie A.B., Zhang D., Dirk L.M.A., Thacker R.R., Pfeiffer J.A.,
Drake J.L., Levy A.A., Butterfield D.A., Buxton J.W., Snyder J.C.
Communication between the maternal testa and the embryo and/or
endosperm affect testa attributes in tomato. Plant Physiol. 2003;
133(1):145-160. DOI 10.1104/pp.103.022632

Ferdinando M.D., Brunetti C., Fini A., Tattini M. Flavonoids as antioxidants
in plants under abiotic stresses. In: Ahmad P., Prasad M. (Eds.)
Abiotic Stress Responses in Plants. New York: Springer, 2012;159-
179. DOI 10.1007/978-1-4614-0634-1_9

Flintham J., Adlam R., Bassoi M., Holdsworth M., Gale M. Mapping
genes for resistance to sprouting damage in wheat. Euphytica. 2002;
126:39-45. DOI 10.1023/A:1019632008244

Glagoleva A.Y., Shoeva O.Y., Khlestkina E.K. Melanin pigment in
plants: current knowledge and future perspectives. Front. Plant Sci.
2020;11:770. DOI 10.3389/fpls.2020.00770

He K., Zhang X., Ren S., Sun J. Deep residual learning for image recognition.
In: Proceedings of the IEEE Conference on Computer Vision
and Pattern Recognition (CVPR), Las Vegas, NV, USA, 2016. IEEE,
2016;770-778. DOI 10.1109/CVPR.2016.90

Huang M., Wang Q.G., Zhu Q.B., Qin J.W., Huang G. Review of seed
quality and safety tests using optical sensing technologies. Seed Sci.
Technol. 2015;43(3):337-366. DOI 10.15258/sst.2015.43.3.16

Jana B.K., Mukherjee S.K. Notes on the distribution of phytomelanin
layer in higher plants – a short communication. J. Pharm. Biol.
2014;4(3):131-132

Jolliffe I.T. Principal Component Analysis. Springer Series in Statistics.
New York: Springer, 2002. DOI 10.1007/b98835

Khojastehnazhand M., Roostaei M. Classification of seven Iranian
wheat varieties using texture features. Expert Syst. Appl. 2022;199:
117014. DOI 10.1016/j.eswa.2022.117014

Kolhar S., Jagtap J. Plant trait estimation and classification studies in
plant phenotyping using machine vision. A review. Inf. Process.
Agric. 2023;10(1):114-135. DOI 10.1016/j.inpa.2021.02.006

Komyshev E.G., Genaev M.A., Afonnikov D.A. Analysis of color and
texture characteristics of cereals on digital images. Vavilovskii Zhurnal
Genetiki i Selektsii = Vavilov Journal of Genetics and Breeding.
2020;24(4):340-347. DOI 10.18699/VJ20.626

Kozłowski M., Górecki P., Szczypiński P.M. Varietal classification of
barley by convolutional neural networks. Biosyst. Eng. 2019;184:
155-165. DOI 10.1016/j.biosystemseng.2019.06.012

Li Z., Guo R., Li M., Chen Y., Li G. A review of computer vision technologies
for plant phenotyping. Comput. Electron. Agric. 2020;176:
105672. DOI 10.1016/j.compag.2020.105672

Neuman M.R., Sapirstein H.D., Shwedyk E., Bushuk W. Wheat grain
colour analysis by digital image processing II. Wheat class discrimination. J. Cereal Sci. 1989;10(3):183-188. DOI 10.1016/S0733-
5210(89)80047-5

Pedregosa F., Varoquaux G., Gramfort A., Michel V., Thirion B.,
Grisel O., Blondel M., Prettenhofer P., Weiss R., Dubourg V., Vanderplas
J., Passos A., Cournapeau D., Brucher M., Perrot M., Duchesnay
E. Scikit-learn: machine learning in Python. J. Mach.
Learn. Res. 2011;12:2825-2830

Ronneberger O., Fischer P., Brox T. U-Net: convolutional networks
for biomedical image segmentation. In: Navab N., Hornegger J.,
Wells W., Frangi A. (Eds.) Medical Image Computing and Computer-
Assisted Intervention – MICCAI 2015. Lecture Notes in
Computer Science. Vol. 9351. Cham: Springer, 2015;234-241. DOI
10.1007/978-3-319-24574-4_28

Sabanci K., Kayabasi A., Toktas A. Computer vision-based method
for classification of wheat grains using artificial neural network.
J. Sci. Food Agric. 2017;97(8):2588-2593. DOI 10.1002/jsfa.8080

Sharma R., Kumar M., Alam M.S. Image processing techniques to
estimate weight and morphological parameters for selected wheat
refractions. Sci. Rep. 2021;11(1):20953. DOI 10.1038/s41598-021-
00081-4

Shoeva O.Yu., Strygina K.V., Khlestkina E.K. Genes determining the
synthesis of flavonoid and melanin pigments in barley. Vavilovskii
Zhurnal Genetiki i Selektsii = Vavilov Journal of Genetics and
Breeding. 2018;22(3):333-342. DOI 10.18699/VJ18.369 (in Russian)

Souza F.H., Marcos-Filho J. The seed coat as a modulator of seed-environment
relationships in Fabaceae. Braz. J. Bot. 2001;24(4):365-
375. DOI 10.1590/S0100-84042001000400002

Tan M., Le Q. EfficientNet: rethinking model scaling for convolutional
neural networks. In: Proceedings of the 36th International Conference
on Machine Learning, Long Beach, California, 9–15 June
2019. ICML, 2019;6105-6114

van der Maaten L., Hinton G. Visualizing data using t-SNE. J. Mach.
Learn. Res. 2008;9(11):2579-2605.

Wang Y.H., Su W.H. Convolutional neural networks in computer vision
for grain crop phenotyping: a review. Agronomy. 2022;12(11):2659.
DOI 10.3390/agronomy12112659

Zykin P.A., Andreeva E.A., Tsvetkova N.V., Voylokov A.V. Anatomical
and image analysis of grain coloration in rye. Preprints. 2020;
2020110530. DOI 10.20944/preprints202011.0530.v1

